# FTIR Spectroscopy Coupled with Principal Component Analysis for Rapid Screening of Melamine Adulteration in Brown Rice Flour

**DOI:** 10.3390/molecules31111912

**Published:** 2026-06-02

**Authors:** Cristina Pintilii, Leonard Mihaly Cozmuta, Zsolt Szakacs, Anca Mihaly Cozmuta

**Affiliations:** Chemistry-Biology Department, Faculty of Science, North University Center of Baia Mare, Technical University of Cluj-Napoca, Victoriei Str. 76, 430122 Baia Mare, Romania; pintiliicristi@yahoo.com (C.P.); mihalyleonard@yahoo.com (L.M.C.); szakacsz@yahoo.com (Z.S.)

**Keywords:** melamine, brown rice flour, FTIR spectroscopy, chemometric analysis, food fraud identification, principal component analysis (PCA), food authenticity, multivariate calibration, second-order derivative spectroscopy

## Abstract

Food adulteration with melamine represents a serious threat to food safety due to its toxic effects and its ability to falsely elevate protein values measured by nitrogen-based methods. Visual inspection and visible reflectance spectroscopy are unsuitable for identifying low-level adulteration. This study evaluates Fourier Transform Infrared (FTIR) spectroscopy combined with chemometric tools for the identification of melamine in brown rice flour adulterated at 0–2.00% (*w*/*w*). Under the tested conditions, no clear FTIR-detectable interactions between melamine and starch or proteins were observed, suggesting that melamine primarily acts as a physical admixture. Characteristic melamine absorption bands were identified at 3466, 3415, 1431, and 810 cm^−1^. Spectral normalization and second-order derivative processing improved sensitivity and enabled quantitative calibration models. The method achieved a limit of detection of 1408 mg/kg. Although this value is above the regulatory threshold of 2.5 mg/kg, the approach provides a rapid, non-destructive screening tool for identifying highly adulterated samples and prioritizing them for confirmatory chromatographic or mass spectrometric analysis. Overall, FTIR spectroscopy combined with chemometric analysis offers an efficient first-line approach for identification of melamine adulteration in brown rice flour.

## 1. Introduction

The deliberate adulteration of food products with non-nutritive or economically motivated adulterants represents a major food safety concern, posing risks to public health and undermining consumer confidence worldwide.

Among such adulterants, melamine has emerged as a critical compound due to its ability to mimic protein content while presenting severe toxicological risks. Melamine (2,4,6-triamino-1,3,5-triazine) is a trimer of cyanamide with 66.67% nitrogen, extensively used in industrial applications, including laminates, adhesives, coatings, molding compounds, dinnerware, and flame retardants [1]. Although not authorized for food use, melamine has been employed to artificially elevate apparent protein content in foods for economic gain [2]. Conventional protein determination methods, such as those by Kjeldahl [3] and Dumas [4], rely on total nitrogen measurements and cannot distinguish protein-derived nitrogen from non-protein nitrogen.

Studies have documented melamine toxicity extensively and reported oxidative stress, activation of NF-κB and NADPH oxidase pathways, excessive reactive oxygen species generation, impaired antioxidant defenses, and cytotoxicity, particularly in renal cells [5,6,7]. It also forms nephrotoxic crystals with cyanuric and uric acids and has been associated with renal injury, reproductive toxicity, neurotoxicity, and potential carcinogenic and hematological effects [8,9].

Researchers have applied advanced detection techniques in powdered milk, such as infrared thermography with convolutional neural networks [10], DNA nanostructure-based biosensors [11], and various optical spectroscopy methods [12,13,14,15]. NIRS combined with multivariate analysis was used for analysis of protein supplements [16]. Researchers applied analytical methods such as HPLC in cereal-based products for detection of melamine and its triazine by-products (ammeline, ammelide, cyanuric acid) in cereals flours and concentrates [17,18], and commercial ELISA for infant formula and wheat-derived products [19]. Studies have reported isotope dilution LC–MS for the determination of melamine and cyanuric acid in catfish, pork, chicken, and pet food [20], whereas HPLC–MS/MS has been employed for the quantification and confirmation of melamine in catfish, trout, tilapia, salmon, and shrimp [21].

Historical reports indicate intentional melamine adulteration in foods and feeds to falsely increase protein content. The first large-scale event occurred in 2007, when melamine and cyanuric acid were detected in wheat gluten and other protein concentrates used in pet foods, causing widespread recalls and severe renal failure in cats and dogs in North America and Europe [22,23]. The most severe human health crisis followed in 2008 in China, where melamine-contaminated diluted milk and infant formula led to ~300,000 urinary tract illnesses and at least six infant deaths [24]. Secondary contamination of animal-derived products, including eggs and meat, via melamine-tainted feed prompted international recalls and trade restrictions [2,25,26]. Collectively, these incidents revealed critical limitations of total nitrogen-based protein assays and highlighted the need for regulatory limits, enhanced surveillance, and development of selective analytical methods for melamine and its analogues.

Brown rice flour, widely used in gluten-free foods, infant formulations, and animal feeds, exhibits considerable variability in protein content and quality depending on cultivar, agronomic conditions, and post-harvest processing [27]. Such variability can lead to nutritionally suboptimal protein levels, highlighting the need for protein reinforcement or fortification strategies. Recognized methods for protein reinforcement are classified based on origin of enrichment. Exogenous reconstitution involves the incorporation of rice protein concentrates or isolates [28], whereas endogenous enrichment increases the relative protein content of rice flour through enzymatic starch degradation [29] or physical starch separation [30]. In contrast, the addition of nitrogen-rich chemical adulterants including melamine, cyanuric acid, ammeline, and ammelide to rice-derived products constitutes an illegal practice [31].

**Figure 1:**
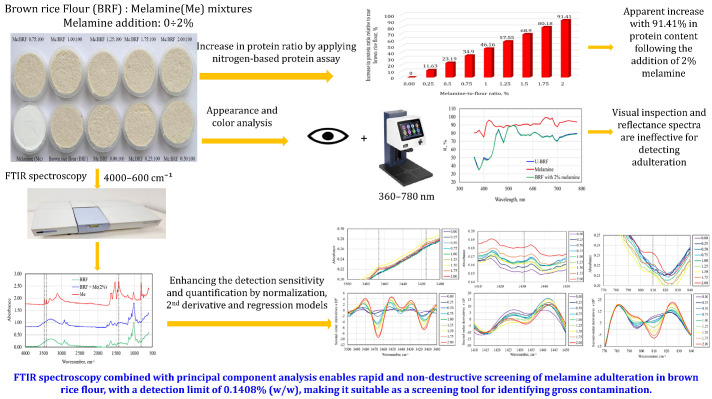
Schematic diagram illustrating the optical setup for FTIR and reflectance measurements.

Although FTIR spectroscopy combined with chemometric analysis has previously been investigated for melamine detection in several food matrices, particularly dairy products and infant formula powders [32,33,34], limited information is available regarding its applicability to brown rice flour. Previous studies using near- and mid-infrared spectroscopy, FTIR-ATR, and FTIR-DRIFT coupled with multivariate analysis demonstrated sensitive detection and classification of melamine-adulterated infant formula samples with minimal sample preparation [33]. FTIR spectroscopy combined with classification approaches such as LDA, PLS-DA, SIMCA, KNN, and CART has also been successfully applied for the discrimination of melamine and cyanuric acid adulteration in powdered infant formulas [34]. However, cereal-based matrices such as brown rice flour differ substantially from dairy systems because of their starch-dominated composition, lower protein content, heterogeneous particulate structure, and different spectral background characteristics, which may contribute to spectral masking effects and reduced detection sensitivity. Brown rice flour represents a particularly relevant matrix because of its widespread use in gluten-free and infant-oriented foods and the potential economic incentive for protein-related adulteration. In addition, previous spectroscopic investigations have focused primarily on detection capability, whereas limited attention has been given to the influence of melamine addition on starch crystallinity, protein secondary structure, spectral variability, and the comparative performance of visible reflectance spectroscopy versus ATR-FTIR approaches in cereal-based systems. Therefore, this study aims not only to evaluate FTIR-based detection of melamine in brown rice flour, but also to assess the structural and chemometric aspects that influence screening performance. Specifically, the study addresses: (i) the impact of melamine addition on calculated protein content; (ii) potential chemical interactions between melamine and starch; (iii) potential interactions of melamine with proteins in brown rice flour; (iv) effects on reflectance spectra and color parameters; and (v) detection and identification of melamine using FTIR spectral features and chemometric tools, including determination of the limit of detection. Figure 1 shows a schematic diagram illustrating the optical setup for FTIR and reflectance measurements.

## 2. Results

### 2.1. Effect of Melamine Addition on Nitrogen-Based Protein Estimation in Brown Rice Flour

The apparent protein content of brown rice flour increased systematically with increasing melamine addition (Table 1), rising from 8.33% in the unadulterated sample to 15.94% at a melamine level of 2.0% (*w*/*w*), despite no actual increase in true protein content. Even at relatively low adulteration levels, such as 0.25% (*w*/*w*), the calculated protein content increased sufficiently to alter the measured nutritional profile. At higher melamine concentrations, the apparent protein values approached those typically associated with protein-enriched cereal ingredients.

### 2.2. Visual Assessment of Brown Rice Flour Samples

**Figure 2:**
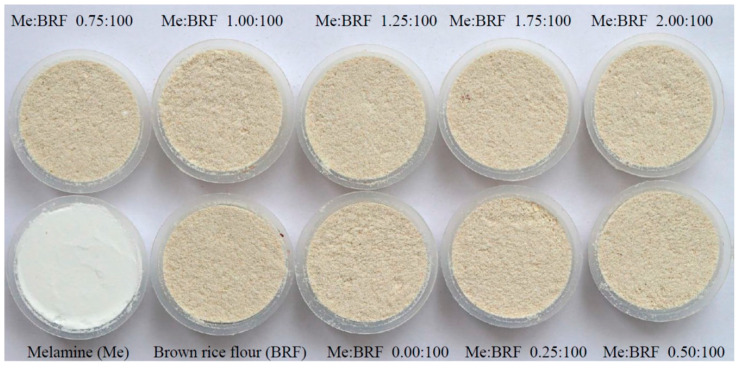
Appearance of melamine, un-adulterated brown rice flour and brown rice flour adulterated with different ratios of melamine.

Visual inspection of the samples (Figure 2) indicated that brown rice flour containing added melamine was indistinguishable from uncontaminated flour at the tested concentrations. All flour-based samples exhibited similar off-white to light beige coloration and a comparable fine powder texture. In contrast, the pure melamine sample displayed a noticeably brighter and more uniform white appearance. No consistent differences in color, homogeneity, or surface characteristics were observed between the control and adulterated flour samples.

### 2.3. Reflectance Spectra

**Figure 3:**
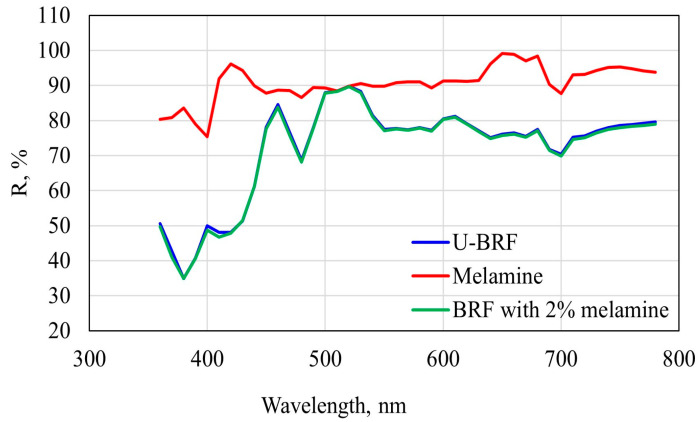
Reflectance spectra of melamine, un-adulterated brown rice flour (U-BRF) and brown rice flour with 2% melamine.

The reflectance spectra (Figure 3) reveal clear differences among unadulterated brown rice flour (U-BRF), pure melamine, and brown rice flour adulterated with 2% melamine, reflecting their distinct chemical and physical characteristics. U-BRF exhibits relatively low reflectance in the visible region (≈350–450 nm), followed by a gradual increase toward longer wavelengths. This is characteristic of cereal flours and can be attributed to light absorption by pigments and organic constituents such as phenolic compounds and proteins. In contrast, melamine shows consistently high reflectance across the entire measured wavelength range (≈380–780 nm), with values generally above 85–90%, consistent with its crystalline structure, high purity, and lack of strongly absorbing chromophores in the visible region [35].

### 2.4. The Impact of Melamine Addition on Starch Crystallinity in Brown Rice Flour

The raw FTIR spectra were normalized, and baseline correction was applied over the 1200–900 cm^−1^ region. Quantitative analysis of starch structures was performed through deconvolution using Gaussian functions, with band center positions determined from the minima of the second-order derivative. The relative crystallinity degree of starch (RCD, %) was calculated as the percentage of the areas corresponding to the crystalline form relative to the total area. FTIR spectra for the investigated rice flour samples were acquired in triplicate. Each spectrum was subjected to deconvolution, and the RCD (%) was calculated. The results (Table 2) are expressed as the mean of three independent determinations, with relative standard deviation (RSD, %) values ranging from 0.53 to 6.1%. Statistical analysis using ANOVA followed by Tukey’s test indicated no significant differences in starch crystallinity among the samples. The correlation coefficient (r = −0.2718, *p* > 0.05) showed no statistically significant relationship between relative crystallinity and melamine content.

### 2.5. The Impact of Melamine Addition on Secondary Structure of Protein in Brown Rice Flour

The secondary structure of proteins was analyzed by spectral deconvolution over the characteristic 1750–1600 cm^−1^ region. The wavenumbers corresponding to the absorption band centers were determined from the minima of the second-order derivative. According to Zhao et al. [34], the protein secondary structure was quantified based on the areas of the Gaussian-fitted distributions corresponding to the following regions: α-helix (1663–1646 cm^−1^), β-sheet (1636–1615 cm^−1^), β-turn (1681–1664 cm^−1^), and random coil (1645–1637 cm^−1^). Table 2 presents the mean values of the component of the secondary structure of protein in analyzed samples along with the results of ANOVA and Tukey’s test for comparative analysis. The results represent the mean of three independent determinations. Data variability, expressed as the RSD (%), ranged from 1.92 to 5.56%, reflecting the quality and reproducibility of the deconvolution process.

### 2.6. Analysis of Data Variability

Before analyzing the possibility of FTIR detecting adulteration of brown rice flour with melamine, an initial assessment of data variability is necessary. For each of the nine samples (as listed in Table 1) with melamine concentrations ranging from 0.25% to 2.00%, three individual FTIR spectra were recorded, replacing the material on the Miracle ATR sample holder each time. Primarily due to differences in the thickness of the material placed on the diamond crystal, the electromagnetic radiation is penetrated, absorbed, and reflected differently, resulting in slightly varying absorbance values. Consequently, for the same material (considered perfectly homogeneous, so that differences cannot arise from compositional variation), distinct FTIR spectra are obtained, exhibiting the same overall shape but with slightly shifted absorbance ranges. Data variability was characterized using the following approach. For each homogeneous sample, three FTIR spectra were recorded, replacing the material on the ATR sample holder between measurements. At each wavenumber (4000–600 cm^−1^), the mean absorbance and standard deviation were calculated from the triplicate spectra. Subsequently, the mean of the mean absorbances and the mean of the standard deviations were computed. Data variability was expressed as the relative standard deviation (RSD, %) of the mean standard deviation relative to the mean of the mean absorbance values. Each of the nine samples was thus associated with a specific measure of individual data variability. The mean of these individual variabilities was 56.43% and was considered as the intragroup variability for the corresponding ANOVA analysis (Table 3). This variability is attributed to the homogeneity of the material and to the precision and accuracy of the FTIR spectroscopic measurement equipment. To eliminate differences caused by varying sample thickness on the ATR sample holder, it is necessary to normalize the raw FTIR spectra. Normalization scales the absorbance values to the range 0–1 for all individual spectra, thereby reducing data variability. In this way, data variability is attributed solely to the homogeneity of the material or sample, without being affected by differences in sample thickness on the ATR crystal. After normalizing the individual spectra and recalculating, variability ranged from 2.38% to 7.04%, with a mean of 4.87% (Table 3).

**Figure 4:**
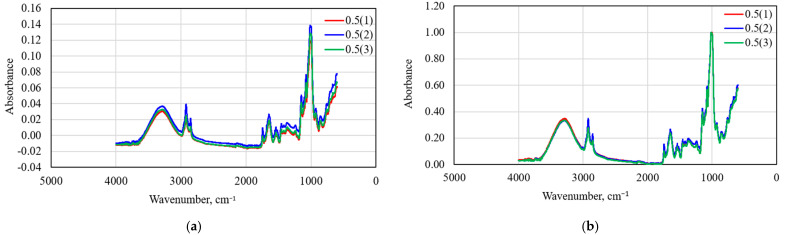
Data variability for raw (**a**) and normalized (**b**) FTIR spectra for the brown rice flour-0.5% melamine mixture.

The addition of melamine to brown rice flour to achieve varying concentrations (0.25–2.00%) is expected to induce a slight increase in data variability due to the presence of differing melamine levels. This variability is considered as intergroup variability. To quantify this effect, the total variability across all 27 FTIR spectra was calculated as 62.59% (Table 3). Subtracting the intragroup variability of 56.43% (corresponding to the mean variability of individual samples) yielded an intergroup variability of 6.17%, which can be attributed to the presence of melamine, representing a 10.93% increase in the initial data variability. For the normalized spectra, total variability was reduced to 6.11% (Table 3). Considering an intragroup variability of 4.87%, the intergroup variability was 1.24%, corresponding to a 25.49% increase due to melamine addition. A comparison of these results, 10.93% for raw spectra versus 25.49% for normalized spectra, indicates that the optimal approach for identifying melamine in brown rice flour mixtures is through analysis of normalized FTIR spectra. As an example, Figure 4a,b show three FTIR spectra recorded for the brown rice flour with the 5% melamine mixture. The data variability for the three raw spectra was 45.83%, whereas normalization reduced variability to 4.88%.

### 2.7. Application of FTIR for the Identification of Melamine Adulteration in Brown Rice Flour

**Figure 5:**
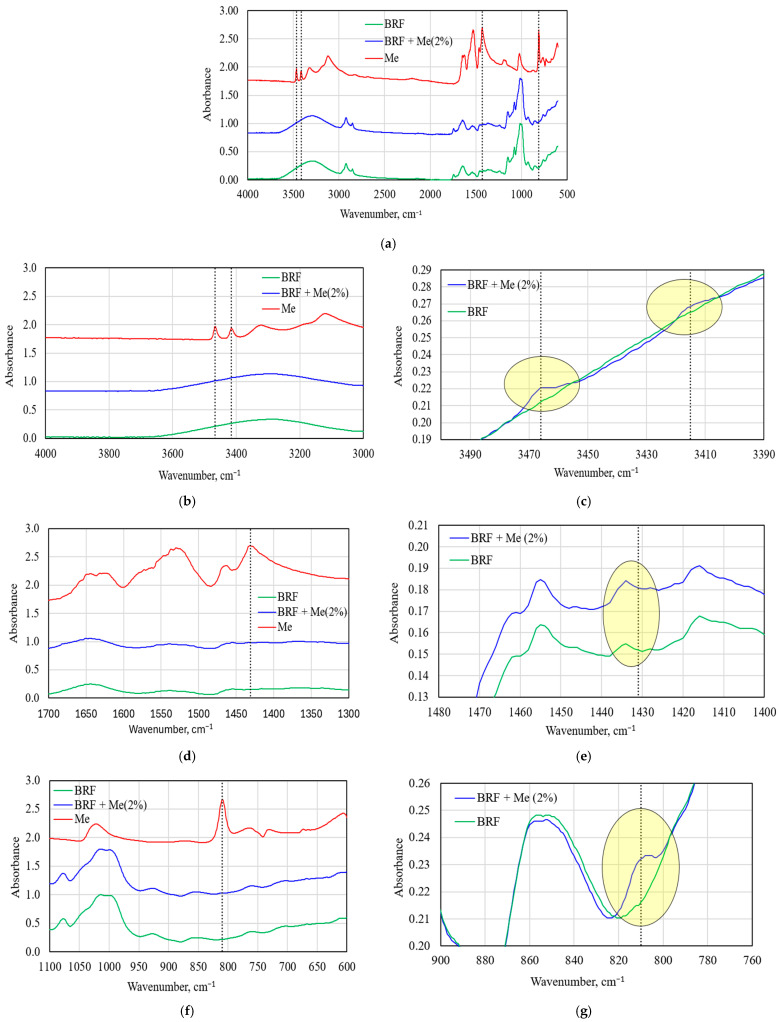
FTIR spectra of brown rice flour (BRF), melamine (Me), and BRF adulterated with 2% melamine: (**a**) raw spectra; (**b**,**c**) spectral region of the 3466 and 3415 cm^−1^ bands; (**d**,**e**) spectral region of the 1431 cm^−1^ band; (**f**,**g**) spectral region of the 810 cm^−1^ band.

Figure 5a shows the FTIR spectra of brown rice flour, melamine, and a mixture of brown rice flour with 2.00% melamine.

In the case of brown rice flour, the following characteristic bands are observed: (i) a broad absorption between 3300 and 3000 cm^−1^ corresponding to O–H stretching vibrations [35]; (ii) bands at 2920–2850 cm^−1^ attributed to C–H stretching vibrations from the aliphatic chains of lipids [36]; (iii) the amide I region at 1700–1600 cm^−1^, associated with C=O stretching of the amide group and minor N–H bending vibration characteristics [37,38,39]; the amide II region at 1600–1500 cm^−1^, corresponding to N–H in-plane bending and C–N stretching vibrations [40]; and the 1200–1000 cm^−1^ region, a characteristic of polysaccharide structures, reflecting stretching vibrations of functional groups of C-O, C-C, and C-O-H and of the glycosidic bond in starch, cellulose, and sugars [40,41].

In the case of melamine, eleven absorption maxima were identified, which can be grouped into three categories: (i) ν(-NH_2_) vibrations and deformation of amino groups at 3466, 3415, 3321, and 3119 cm^−1^ [42,43]; (ii) δ(NH_2_) stretching vibrations at 1644 and 1626 cm^−1^, ν(CN) + δ (NH_2_) at 1530 cm^−1^, and ring deformation and ρ(NH_2_) at 1022 cm^−1^ [42]; (iii) in-plane vibrations of the triazine ring and deformations of the ν(CN) + δ (NH_2_) groups at 1463 and 1431 cm^−1^ [42]; and (iv) the out-of-plane vibrations of the triazine ring at 810 cm^−1^ [42], corresponding to the so-called “golden vibration” characteristic of melamine, are strong and intense, highly reproducible, spectrally well isolated with minimal interference from common polymers or organic compounds, and highly specific to melamine and related triazine derivatives, thereby making this band a reliable diagnostic marker for their identification. To identify the presence of melamine in brown rice, the FTIR spectrum was analyzed, focusing on absorption maxima characteristic of melamine.

**Figure 6:**
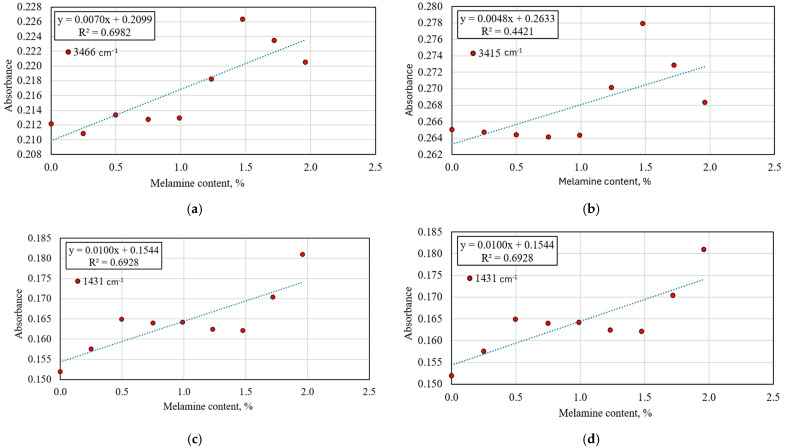
Calibration curves showing the variation in absorbance as a function of melamine concentration in brown rice flour–melamine mixtures at the wavenumbers 3466 cm^−1^ (**a**), 3415 cm^−1^ (**b**), 1431 cm^−1^ (**c**), and 810 cm^−1^ (**d**).

Three characteristic melamine-related spectral regions were identified in the mixture spectrum. The absorption bands at 3466 and 3415 cm^−1^ are shown in Figure 5b,c. The spectral region corresponding to 1431 cm^−1^ is presented in Figure 5d,e. The characteristic band at 810 cm^−1^ is shown in Figure 5f,g. At 1431 cm^−1^, although no distinct absorption maximum is observed, the difference in absorbance values between unadulterated and adulterated brown rice flour is maximal because melamine exhibits significantly higher absorbance at this wavenumber than brown rice flour. Consequently, even at low melamine concentrations, the absorbance of the mixtures is markedly increased (Figure 5f,g). From the nine averaged FTIR spectra of brown rice flour and mixtures with melamine within the concentration range of 0.25–2.00%, absorbance values corresponding to the four previously identified wavenumbers were extracted, and calibration curves depicting the variation in absorbance as a function of melamine concentration were constructed (Figure 6a–d). In all four cases, the correlation coefficients were relatively low, ranging from 0.3526 to 0.8356. Although differences in the FTIR spectra caused by the presence of melamine were observed, these differences were small. Consequently, while the method can reliably identify adulteration, it does not allow for accurate and precise quantitative determination of melamine content.

**Figure 7:**
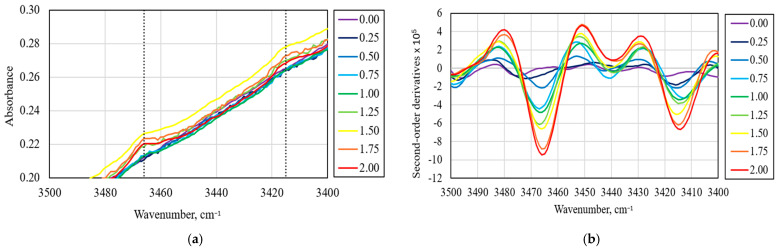

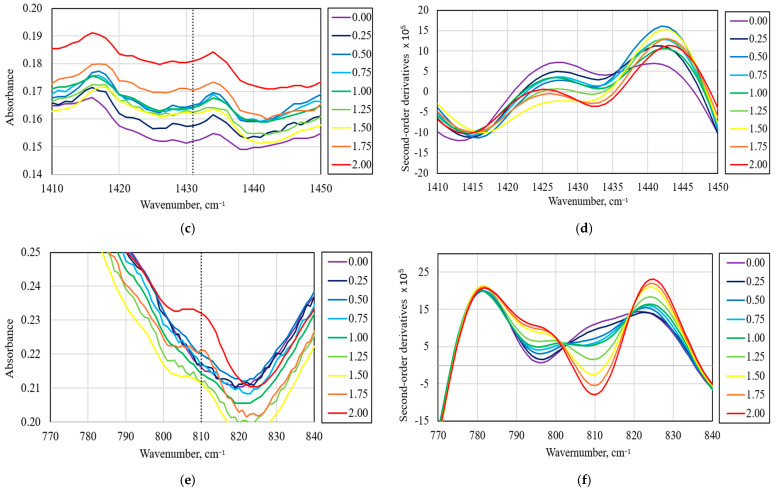
Changes in FTIR spectra and second-order derivatives of brown rice flour (BRF) adulterated with melamine at different concentrations: (**a**) FTIR spectra in the 3466 and 3415 cm^−1^ region; (**b**) corresponding second-order derivative spectra; (**c**) FTIR spectra in the 1431 cm^−1^ region; (**d**) corresponding second-order derivative spectra; (**e**) FTIR spectra in the 810 cm^−1^ region; (**f**) corresponding second-order derivative spectra.

**Figure 8:**
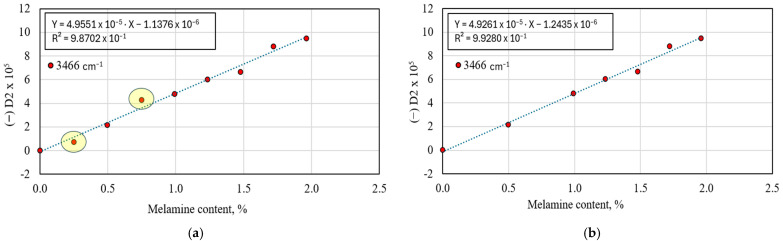

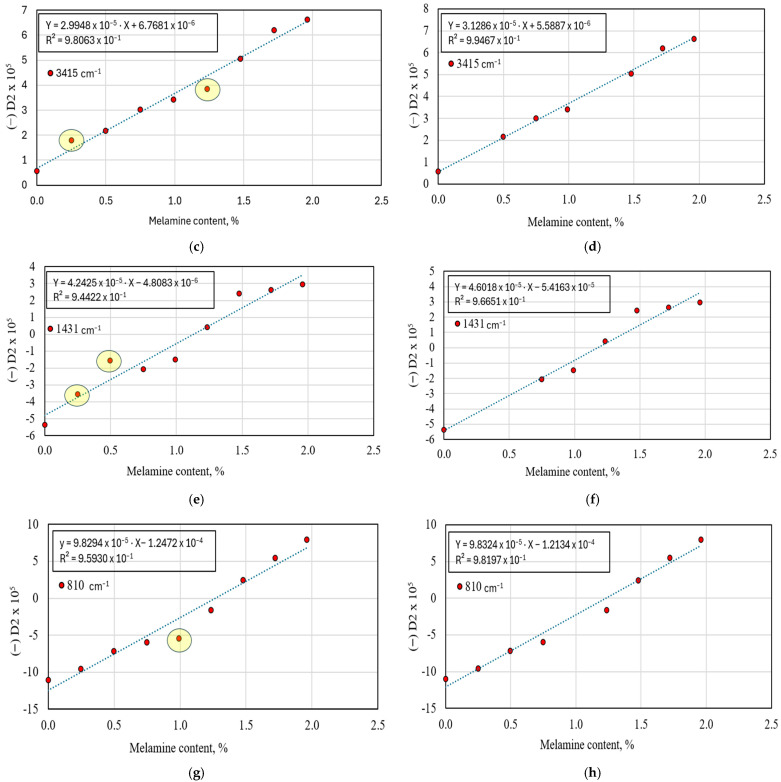
Calibration curves for the determination of melamine in brown rice flour using second-order derivative spectra: (**a**) 3466 cm^−1^ region; (**b**) 3466 cm^−1^ region after exclusion of outliers; (**c**), 3415 cm^−1^ region; (**d**) 3415 cm^−1^ region after exclusion of outliers; (**e**) 1431 cm^−1^ region; (**f**) 1431 cm^−1^ region after exclusion of outliers; (**g**) 810 cm^−1^ region; (**h**) 810 cm^−1^ region after exclusion of outliers.

For the quantitative assessment of melamine in adulterated brown rice flour samples, the second-order derivative approach as described by Pintilii et al. (2025) [44] was employed. Briefly, the second-order derivatives were calculated from the normalized FTIR spectra using the Savitzky–Golay algorithm with a third-degree polynomial and a 25-point window. Since derivatization enhances the resolution of both the analytical signal and the background noise, the latter was minimized by smoothing the derivative spectra using the same Savitzky–Golay parameters (third-degree polynomial, 25-point window). Figure 7 comparatively presents the variations in absorbance values and in the second-order derivatives for the four characteristic spectral regions that enable the identification of melamine. Based on these derivatives, calibration curves were constructed, allowing the quantification of melamine in adulterated brown rice flour samples. Figure 8a,c,e,g present the calibration curves employed to determine melamine content in adulterated samples, obtained from second-order derivatives. Unit derivative absorbances, corresponding to a 1% melamine content, were used to evaluate the detection limits of the linear regression models and were subjected to outlier tests. The following methods were applied: box-plot, Grubbs, Chauvenet, Nalimov, Romanowski, Irwin, Q, and Dean-Dixon. Following the exclusion of invalid values, the calibration curves were reconstructed, and the detection limits were subsequently reassessed (Figure 8b,d,f,h).

In all cases, the removal of inappropriate data resulted in a decrease in the detection limit for melamine. The optimal model was identified as Model 7, exhibiting the lowest detection limit of 0.1408% melamine in adulterated brown rice flour. At the detection limit, the apparent protein concentration in the adulterated brown rice flour increases from the actual value of 8.33% to 8.88% (Table 4: Characterization of the linear regression models obtained before and after outlier exclusion, presenting only the essential parameters relevant to the study objectives). The complete set of model parameters is provided in Section S2 in the Supplementary Materials.

### 2.8. Principal Component Regression Integrated with Principal Component Analysis

Principal component analysis (PCA) was applied to investigate the adulteration of brown rice flour with melamine. For this purpose, the analysis focused on the following wavenumber regions: 3466 cm^−1^ (3480–3450), 3415 cm^−1^ (3430–3400), 1431 cm^−1^ (1440–1425), and 810 cm^−1^ (825–790), yielding a total of 114 data points. Corresponding absorbance values and second-order derivative values were extracted and subjected to PCA. Table 5 presents the characterization of data variability explained by the identified principal components. Correlation coefficients between the principal component values and the melamine concentrations in the mixtures were calculated to evaluate their quantitative significance.

**Figure 9:**
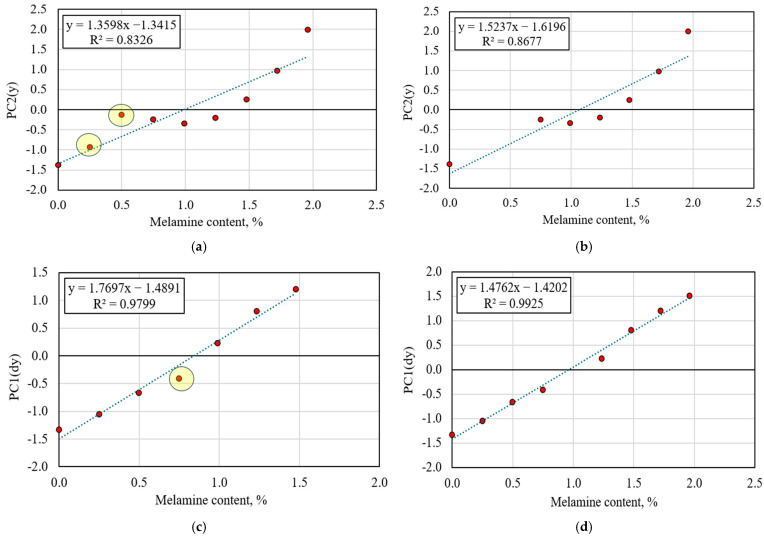
Calibration curves of principal components versus melamine content (%): PC2 from absorbance values and PC1 from second-order derivative values, shown before (**a**,**c**) and after (**b**,**d**) exclusion of outliers.

Figure 9 presents the calibration curve *PC*2 = *f*(*Melamine content*, %) corresponding to the analysis of absorbance values and the calibration curve *PC*1 = *f*(*Melamine content*, %) corresponding to the analysis of second-order derivative values, both before and after the exclusion of outlier data.

Table 6 reports the characterization of these regression models, showing only the essential parameters, with the complete set provided in Section S2 of the Supplementary Materials.

## 3. Discussion

### 3.1. Effect of Melamine Addition on Nitrogen-Based Protein Estimation in Brown Rice Flour

The observed increase in apparent protein content reflects the contribution of melamine-derived nitrogen to total nitrogen measurements, demonstrating the susceptibility of nitrogen-based protein estimation methods to intentional adulteration. Since protein labeling is commonly based on total nitrogen determination using the Kjeldahl or Dumas procedure [3,4], the additional nitrogen introduced by melamine is directly converted into artificially elevated protein values. From a regulatory and consumer protection perspective, these findings are significant. Brown rice flour is frequently used in gluten-free products, infant formulations, and specialized dietary foods, where protein content is a critical nutritional parameter. Artificial inflation of protein values through non-protein nitrogen adulteration may therefore enable misleading nutritional claims without any genuine improvement in protein quality, undermining the integrity of food labeling and posing particular concerns for vulnerable populations. Overall, the results indicate that reliance solely on total nitrogen-based protein determination is insufficient to ensure protein authenticity in cereal-based products. This highlights the need for complementary analytical approaches capable of detecting non-protein nitrogen adulterants, as well as the importance of rapid screening methods to identify potential adulteration prior to confirmatory analysis.

### 3.2. Visual Assessment of Brown Rice Flour Samples

The absence of discernible visual differences between uncontaminated and melamine-adulterated samples demonstrates the limitations of sensory-based evaluation for detecting this type of adulteration. Even at moderate inclusion levels, melamine does not significantly alter the macroscopic appearance of brown rice flour, rendering visual inspection ineffective as a screening tool. This finding underscores the necessity of applying analytical techniques for reliable detection and quantification, particularly in quality control settings where rapid and accurate identification of adulterants is critical.

### 3.3. Reflectance Spectra

The reflectance spectra of unadulterated brown rice flour (U-BRF) and brown rice flour adulterated with 2% melamine exhibit very similar spectral profiles across the entire visible wavelength range (≈380–780 nm), with no pronounced or distinct spectral features that would clearly differentiate the two samples. Both spectra show comparable reflectance trends, characterized by lower reflectance at shorter wavelengths followed by a gradual increase toward the red region, which is typical of cereal flours and reflects their similar physical structure and light-scattering behavior. Although melamine alone displays consistently high reflectance values throughout the measured range, its presence at a 2% level does not produce a statistically discernible alteration in the overall reflectance pattern of the flour matrix. This suggests that, at low adulteration levels, the strong scattering and absorption characteristics of brown rice flour dominate the composite spectrum, effectively masking the spectral contribution of melamine. These results indicate that visible reflectance spectroscopy, when used alone, may have limited sensitivity for identifying low-level melamine adulteration in brown rice flour and highlight the need for complementary analytical or chemometric approaches to improve discrimination and detection capability.

### 3.4. The Impact of Melamine Addition on Starch Crystallinity in Brown Rice Flour

Quantitative analysis was based on previously reported band assignments, where the spectral regions at 1047 and 995 cm^−1^ are associated with crystalline starch structures, while the band at 1020 cm^−1^ corresponds to the amorphous phase [28].

### 3.5. The Impact of Melamine Addition on Secondary Structure of Protein in Brown Rice Flour

Overall, the data indicate that the secondary structure of proteins in brown rice flour is generally unaffected by melamine addition. No statistically significant differences were observed for the α-helix and β-sheet structures as a function of melamine content. For the β-turn and random coil structures, the minor variations can be attributed to the inherent variability among the individual samples. In addition, the minor variations observed in the β-turn and random coil content of proteins may arise from several well-recognized factors associated with FTIR analysis and protein structure. First, the amide I region (1700–1600 cm^−1^) comprises overlapping contributions from multiple secondary structural motifs, including α-helix, β-sheet, turns, and random coil. This spectral overlap can lead to small differences in the areas assigned to β-turn and random coil structures during deconvolution, even when overall protein structure is consistent across samples [45]. Second, the C=O stretching vibrations responsible for the amide I band are highly sensitive to the local hydrogen-bonding environment. Minor variations in hydration, intermolecular interactions, or the flour matrix environment can subtly shift vibrational frequencies and influence the quantification of specific secondary structure components [46]. Finally, deconvolution procedure is subject to methodological variability, as baseline selection, peak shape assumptions, and fitting constraints which can slightly alter the calculated areas of overlapping bands. Therefore, these factors, rather than the presence of melamine, likely account for the small differences observed in β-turn and random coil structures across the samples.

The lack of detectable changes in starch structure and protein secondary structure upon melamine addition further demonstrates that melamine is physically mixed within the flour matrix without inducing conformational modifications of native starch and proteins. From an analytical perspective, this result enhances detection reliability by confirming that FTIR-based discrimination is driven by melamine-specific absorption features rather than starch or protein structural perturbations, reducing the risk of false positives associated with matrix-induced spectral changes.

### 3.6. Analysis of Data Variability

The variability observed among replicate FTIR measurements was primarily associated with experimental factors intrinsic to FTIR analysis, including sample positioning, contact pressure between the sample and the ATR crystal, and local heterogeneity of the starch-rich matrix. Spectral normalization was therefore essential to minimize intensity-related variability and to ensure comparability between measurements. Without normalization, variations in overall absorbance intensity could obscure subtle melamine-related spectral features and compromise the robustness of subsequent qualitative and quantitative analyses. In the context of routine screening, where samples are handled by different operators and analyzed under variable conditions, normalization represents a critical preprocessing step to ensure that discrimination and modeling are driven by chemical composition rather than experimental artefacts.

### 3.7. Application of FTIR for the Identification of Melamine Adulteration in Brown Rice Flour

The detection limit of 0.14% (*w*/*w*), corresponding to 1408 mg/kg of melamine, is substantially higher than the maximum level of 2.5 mg/kg established by Regulation (EU) 2023/915. The study of Chue et al. [14] reported accuracies exceeding 90% for the quantification of adulteration with melamine (>100 mg/L), urea (>0.5 g/L), sucrose (>0.2%), water (>5%), and milk powder (>50%). A lower detection limit of 0.0001% (1 ppm) in infant formula powder was reported by Mauer et al. (2009) [33]. The higher detection limit obtained in the present study, compared with values reported in previous studies, may be attributed, at least in part, to differences in sample matrix complexity. Brown rice flour presents a more heterogeneous structure, with greater variability in starch composition, and light-scattering properties, which can reduce spectral resolution and affect model sensitivity in FTIR-based chemometric analysis. These matrix effects are well known to influence the performance of infrared spectroscopic methods in solid food systems. Although methods such as LC-MS/MS and LTP-MS/MS provide very low melamine detection limits (down to 250 ppb and 6 ppb, respectively) [33], they generally require longer analysis times (up to 2–3 h) and more complex sample preparation procedures, whereas the FTIR method enabled rapid detection within minutes. Therefore, FTIR cannot be regarded as a stand-alone method for regulatory compliance assessment. However, evidence from previous food fraud incidents indicates that intentional melamine adulteration has typically involved concentrations far exceeding the legal limit, often reaching several hundreds or thousands of mg/kg in products like powdered infant formula, liquid milk and yogurt, powdered milk products, confectionary products, nondairy creamer, and animal feed [24]. Within this risk-based framework for official food controls, FTIR demonstrates suitability as a rapid first-tier screening tool for the identification of gross contamination. The method offers practical advantages, including minimal sample preparation, rapid analysis, non-destructive measurement, and high-throughput capability, which are particularly relevant for routine monitoring activities. Accordingly, FTIR can be employed as a preliminary screening approach, with samples classified as suspect subsequently subjected to confirmatory analysis using more sensitive chromatographic [47] or mass spectrometry techniques [21,33] capable of meeting the performance requirements for enforcement purposes, in line with the principles of Regulation (EU) 2023/915 [48].

### 3.8. Principal Component Regression Integrated with Principal Component Analysis

For the absorbance data, the second principal component (PC2) exhibited the highest correlation (*r* = 0.9125), explaining 36.20% of the total variability (Table 5), which can be attributed to changes in melamine concentration. In contrast, for the second-order derivative data, the first principal component (PC1) showed the strongest correlation (*r* = −0.9904), accounting for 94.27% of the variability, which directly reflects the variation in melamine content. These findings demonstrate that the optimal approach for the quantitative determination of melamine adulteration is based on the analysis of second-order derivative spectra.

Simple linear regression models were also considered between the principal component scores and the melamine concentrations in the adulterated mixtures, following the same procedure as described previously. As the data in Table 6 indicate, the removal of outlier data led to a decrease in the detection limits. The lowest detection limit obtained was 0.1667%, which is slightly higher than the previously established detection limit of 0.1408% based on second-order derivative values.

## 4. Materials and Methods

### 4.1. Reagents

Melamine powder (99% purity, crystalline) was purchased from Sigma-Aldrich Co., Ltd. (St. Louis, MO, USA) and stored in a sealed container at room temperature in a desiccator to protect it from moisture.

### 4.2. Raw Materials

Whole brown rice (SC Bio Navis, Dover, DE, USA) was purchased from a local market. Table S1 presents its proximate composition. Prior to processing, the grains were visually examined to remove broken kernels and extraneous material. The rice was milled using a laboratory grinder (Biovita ME-1, Cluj Napoca, Romania) and subsequently sieved to obtain a particle size below 25 μm.

### 4.3. Sample Formulation

Brown rice flour samples were adulterated with melamine at predefined concentration levels ranging from 0 to 2.00% (*w*/*w*). The components were accurately weighed and homogenized by repeated inversion to ensure uniform distribution of components prior to spectral acquisition. All powders and mixtures were stored in airtight containers at room temperature under dry conditions to prevent moisture uptake and physicochemical changes prior to analysis. Table 1 provides the detailed formulation of each mixture. The melamine concentration range investigated in this study (0–2.00% *w*/*w*) was selected to reflect realistic food fraud scenarios involving intentional adulteration, rather than accidental contamination. Historical food fraud incidents have shown that melamine has often been added at relatively high levels, frequently reaching several hundreds or thousands of mg/kg [24], in order to substantially increase the apparent protein content measured by nitrogen-based analytical methods. To minimize systematic bias and potential instrumental drift, all samples were analyzed in randomized order during both reflectance spectroscopy and FTIR measurements. The sequence of sample analysis was randomized prior to data acquisition, and replicate measurements were performed by removing and repositioning the sample on the ATR crystal between scans. This procedure ensured that any variability arising from sample handling, contact pressure, or instrumental fluctuations was distributed randomly across melamine concentration levels and did not confound the observed spectral differences.

### 4.4. Reflectance Spectra

Spectral differences among rice flour samples were assessed by examining their reflectance spectra, with the objective of evaluating the sensitivity of this approach for the detection of low-level melamine adulteration. Spectral measurements were collected over the 360–780 nm wavelength interval, with each sample analyzed in ten replicate scans at 1 nm increments. A YL4560 non-contact bench spectrophotometer (Shenzen ThreeNH Technology Co., Ltd., Shenzen, China) was used for data acquisition. Analysis of reflectance spectra enables characterization of pigment profiles and identification of potential chemical modifications resulting from melamine incorporation.

### 4.5. FTIR Analysis

The FTIR absorbance spectra of the rice flour samples were acquired using a PerkinElmer BX2 spectrometer (Shelton, CT, USA) equipped with a Pike Miracle diamond ATR crystal. Spectra were collected over the wavenumber range of 4000–600 cm^−1^ with a spectral resolution of 4 cm^−1^, a scanning speed of 0.3 cm/s, and a signal-to-background ratio of 15,000:1, averaging 50 scans per sample. Spectral analysis was primarily focused on assessing alterations in the ordered structure of starch and the secondary structure of proteins, with particular emphasis on the regions corresponding to the 1200–900 cm^−1^ range for starch and the amide I band (1600–1750 cm^−1^) for protein. Spectrum v.5.3.1 software (PerkinElmer, 2009–23384, Shelton, CT, USA) was used for instrument control and for conducting the measurements.

### 4.6. Statistical Analysis

Experiments were performed in triplicate, and results are expressed as the mean ± standard deviation. Statistical differences were evaluated using one-way analysis of variance (ANOVA) followed by Tukey’s post hoc test, with significance set at *p* < 0.05. Principal component analysis (PCA) was conducted to assess differences among brown rice flour samples using Statistica 7.0 (StatSoft, Tulsa, OK, USA).

## 5. Conclusions

This study demonstrates that Fourier Transform Infrared spectroscopy combined with chemometric analysis represents a viable rapid screening approach for identifying melamine adulteration in brown rice flour. Traditional screening methods, such as visual inspection and reflectance spectroscopy, were ineffective even at melamine concentrations as high as 2% (*w*/*w*), highlighting the necessity of molecular-level analytical techniques. Nitrogen-based protein estimation methods were highly susceptible to falsification by melamine, resulting in substantial overestimation of apparent protein content without any real nutritional benefit. These findings confirm that total nitrogen assays alone are inadequate for verifying protein authenticity in cereal-based products and underscore the need for selective analytical strategies.

FTIR analysis revealed that melamine does not chemically interact with either starch or proteins in brown rice flour. The absence of significant changes in starch crystallinity and protein secondary structure indicates that melamine incorporation results in a purely physical mixture rather than chemical modification of the matrix. This finding supports the reliability of FTIR spectral features associated specifically with melamine for adulteration detection. Normalization of FTIR spectra was critical for minimizing instrumental and sampling variability and for enhancing sensitivity to compositional differences. The use of second-order derivative spectra, coupled with Savitzky–Golay smoothing, significantly improved discrimination and enabled robust calibration models for melamine quantification. Although this detection limit is above the regulatory limit of 2.5 mg/kg, FTIR provides a fast and non-destructive screening tool particularly suited to identifying gross contamination and prioritizing suspect samples for confirmatory chromatographic or mass spectrometric analysis. The method is especially suitable for integration into routine incoming raw material screening and import control programs, facilitating rapid decision-making and enhancing supply chain oversight.

Overall, FTIR spectroscopy, combined with normalization, second-order derivatives, and multivariate analysis, offers an efficient and cost-effective approach for rapid screening of melamine adulteration in cereal-based foods. Its principal limitation is the relatively high detection limit, which restricts sensitivity at low adulteration levels, making it unsuitable as a standalone tool for regulatory compliance. Future work should focus on improving sensitivity through advanced chemometric and machine-learning approaches, integrating FTIR with complementary spectroscopic techniques, validating the method using commercially sourced samples and naturally contaminated matrices, and extending validation to diverse cereal matrices. By positioning FTIR as a high-throughput, first-tier screening method, this study highlights its potential to substantially enhance industrial surveillance, fraud detection efficiency, and quality control in cereal-based food production.

## Figures and Tables

**Table 1:** Impact of melamine addition on the calculated protein content of brown rice flour samples.

Melamine-to-Flour Ratio, %	Nitrogen Content in Brown Rice Flour, g	Calculated Nitrogen Contribution of Melamine, g	Total Nitrogen Content, G	Calculated Protein Content, %	Increase in Protein Ratio Relative to Raw Brown Rice Flour, %
0.00	1.4000	0.0000	1.4000	8.33	0.0000
0.25	1.3965	0.1663	1.5628	9.30	11.63
0.50	1.3930	0.3317	1.7247	10.260	23.19
0.75	1.3895	0.4991	1.8886	11.24	34.90
1.00	1.3861	0.6601	2.0462	12.18	46.16
1.25	1.3827	0.8230	2.2058	13.12	57.55
1.50	1.3793	0.9852	2.3645	14.07	68.90
1.75	1.3759	1.1466	2.5225	15.01	80.18
2.00	1.3725	1.3072	2.6797	15.94	91.41

The nitrogen content in melamine was calculated from its molar mass and the added amount. For unadulterated and adulterated brown rice flour samples, nitrogen content was estimated by dividing the protein content by 5.95.

**Table 2:** Impact of melamine addition on the relative crystallinity degree (RCD, %) of starch and secondary structure of protein in brown rice flour samples.

Melamine-to-Flour Ratio, %	Relative Crystallinity Degree of Starch, RCD, %	Secondary Structure of Protein, %			
		α-Helix	β-Sheet	β-Turn	Random Coil
0.00	58.90 ± 1.30 ^a^	22.16 ± 0.78 ^a^	37.74 ± 0.68 ^a^	22.62 ± 0.18 ^ab^	17.48 ± 0.15 ^ab^
0.25	58.73 ± 3.46 ^a^	22.22 ± 0.53 ^a^	37.53 ± 0.30 ^a^	22.72 ± 0.10 ^a^	17.53 ± 0.13 ^ab^
0.50	56.23 ± 0.30 ^a^	22.35 ± 0.68 ^a^	37.98 ± 0.62 ^a^	22.02 ± 0.25 ^c^	17.66 ± 0.07 ^a^
0.75	58.04 ± 3.42 ^a^	22.28 ± 0.44 ^a^	37.74 ± 0.28 ^a^	22.60 ± 0.17 ^abc^	17.39 ± 0.10 ^a^
1.00	57.28 ± 1.11 ^a^	22.38 ± 0.76 ^a^	37.86 ± 0.29 ^a^	22.59 ± 0.28 ^abc^	17.18 ± 0.13 ^b^
1.25	57.56 ± 3.08 ^a^	22.39 ± 0.95 ^a^	37.74 ± 0.36 ^a^	22.36 ± 0.19 ^abc^	17.51 ± 0.16 ^ab^
1.50	56.36 ± 0.92 ^a^	22.19 ± 0.77 ^a^	37.82 ± 0.66 ^a^	22.53 ± 0.26 ^abc^	17.47 ± 0.09 ^ab^
1.75	57.65 ± 3.51 ^a^	22.64 ± 0.50 ^a^	37.90 ± 0.32 ^a^	22.08 ± 0.21 ^bc^	17.38 ± 0.14 ^ab^
2.00	58.37 ± 0.66 ^a^	22.25 ± 0.98 ^a^	37.65 ± 0.73 ^a^	22.58 ± 0.16 ^abc^	17.52 ± 0.17 ^ab^

Data are presented as mean ± standard deviation (n = 3). Values with different letters a, b, and c in the same column are significantly different (*p* < 0.05).

**Table 3:** Analysis of data variability.

	Raw Spectra			Normalized Spectra		
	Average	Stdev	RSD, %	Average	Stdev	RSD, %
Average (intragroup)	0.00	0.00	56.43	0.16	0.01	4.87
stdev	0.00	0.00	34.58	0.00	0.00	1.75
RSD (%)	22.65	71.04	61.28	2.69	38.37	36.02
Total (intergroup)	0.01	0.00	62.59	0.16	0.01	6.11
Differences between intergroup and intragroup			6.17			1.24
Δ (%)			10.93			25.49

**Table 4:** Characterization of linear regression models obtained before and after the exclusion of outlier data.

	Model	M1	M2	M3	M4	M5	M6	M7	M8	M9
	Wavenumber, cm^−1^	3466	3466	3415	3415	1431	1431	1431	810	810
1. Simple linear regression model										
1	a	−1.14 × 10^−6^	−1.24 × 10^−6^	6.77 × 10^−6^	5.59 × 10^−6^	−4.81 × 10^−5^	−5.15 × 10^−5^	−5.42 × 10^−5^	−1.25 × 10^−5^	−1.21 × 10^−5^
2	b	4.96 × 10^−5^	4.93 × 10^−5^	2.99 × 10^−5^	3.13 × 10^−5^	4.24 × 10^−5^	4.43 × 10^−5^	4.60 × 10^−5^	9.83 × 10^−5^	9.83 × 10^−5^
3	r	0.9935	0.9964	0.9903	0.9973	0.9717	0.9831	0.9831	0.9794	0.9909
4	p(r)	7.28 × 10^−8^	1.50 × 10^−6^	2.97 × 10^−7^	7.04 × 10^−7^	1.22 × 10^−5^	1.18 × 10^−5^	7.05 × 10^−5^	4.02 × 10^−6^	1.84 × 10^−6^
5	LD	0.4297	0.1408	0.5814	0.1484	0.7040	0.6036	0.2948	0.4406	0.2641
6	SSE	1.16 × 10^−10^	5.04 × 10^−11^	6.38 × 10^−11^	1.55 × 10^−11^	3.83 × 10^−10^	2.26 × 10^−10^	1.91 × 10^−10^	1.48 × 10^−9^	6.39 × 10^−10^
7	SEE	4.08 × 10^−6^	3.18 × 10^−6^	3.02 × 10^−6^	1.76 × 10^−6^	7.40 × 10^−6^	6.14 × 10^−6^	6.18 × 10^−6^	1.45 × 10^−5^	1.03 × 10^−5^
2. Cross-validation method (CVM)										
8	SSEcv	1.78 × 10^−10^	9.16 × 10^−11^	1.0 × 10^−10^	2.65 × 10^−11^	6.17 × 10^−10^	3.88 × 10^−10^	3.31 × 10^−10^	2.41 × 10^−9^	1.14 × 10^−9^
9	SEEcv	5.04 × 10^−6^	4.28 × 10^−6^	3.78 × 10^−6^	2.30 × 10^−6^	9.39 × 10^−6^	8.04 × 10^−6^	8.14 × 10^−6^	1.85 × 10^−6^	1.38 × 10^−5^
3. ANOVA analysis of the regression model										
10	F	5.32 × 10^2^	6.90 × 10^2^	3.54 × 10^2^	9.34 × 10^2^	1.19 × 10^2^	1.73 × 10^2^	1.44 × 10^2^	1.65 × 10^2^	3.27 × 10^2^
11	p(ANOVA)	7.28 × 10^−8^	1.50 × 10^−6^	2.97 × 10^−7^	7.04 × 10^−7^	1.22 × 10^−5^	1.18 × 10^−5^	7.05 × 10^−5^	4.02 × 10^−5^	1.84 × 10^−5^
4. Testing of the regression model coefficients										
12	p(a)	6.65 × 10^−1^	6.30 × 10^−1^	8.40 × 10^−3^	7.02 × 10^−3^	1.52 × 10^−5^	1.66 × 10^−5^	1.19 × 10^−4^	2.35 × 10^−6^	1.51 × 10^−6^
13	p(b)	7.28 × 10^−8^	1.50 × 10^−6^	2.97 × 10^−7^	7.04 × 10^−7^	1.22 × 10^−5^	1.18 × 10^−5^	7.05 × 10^−5^	4.02 × 10^−6^	1.84 × 10^−6^

Simple linear regression model: a, b: Intercept and slope of the regression line, r, p(r): Correlation coefficient, LD: Detection limit of melamine, SSE, SEE: Sum of squares of residuals and standard error of estimate. Cross-validation: SSEcv, SEEcv: Same as above, calculated during cross-validation to evaluate predictive accuracy. ANOVA for regression: F, p(ANOVA): F-statistic and associated *p*-value testing model significance. Testing regression coefficients: p(a), p(b): t-statistics and *p*-values for intercept and slope.

**Table 5:** Characterization of data variability explained by the identified principal components.

Absorbances	Eigenvalue	% Total Variance	Cumulative Eigenvalue	Cumulative, %
1	1.91 × 10^−3^	56.5883	1.91 × 10^−3^	56.5883
2	1.22 × 10^−3^	36.2009	3.13 × 10^−3^	92.7892
3	2.14 × 10^−4^	6.3546	3.34 × 10^−3^	99.1438
4	9.49 × 10^−6^	0.2817	3.35 × 10^−3^	99.4255
5	7.66 × 10^−6^	0.2275	3.36 ×10^−3^	99.6530
6	5.69 × 10^−6^	0.1688	3.36 × 10^−3^	99.8218
7	3.26 × 10^−6^	0.0967	3.37 × 10^−3^	99.9186
8	2.74 × 10^−6^	0.0814	3.37 × 10^−3^	100.0000
Second-order derivatives	Eigenvalue	% Total variance	Cumulative Eigenvalue	Cumulative, %
1	7.29 × 10^−8^	94.2677	7.29 × 10^−8^	94.2677
2	2.48 × 10^−9^	3.2091	7.54 × 10^−8^	97.4767
3	1.36 × 10^−9^	1.7647	7.68 × 10^−8^	99.2415
4	3.15 × 10^−10^	0.4072	7.71 × 10^−8^	99.6487
5	1.61 × 10^−10^	0.2081	7.72 × 10^−8^	99.8568
6	1.11 × 10^−10^	0.1432	7.73 × 10^−8^	100.0000

**Table 6:** The characterization of these regression models associated with PCA.

	Model	M1	M2	M3	M4	M5
		Absorbances	Absorbances	Absorbances	Second-Order Derivatives	Second-Order Derivatives
		PC2 = f(C)	PC2 = f(C)	PC2 = f(C)	PC1 = f(C)	PC1 = f(C)
1. Simple linear regression model						
1	a	−1.34	−1.50	−1.62	−1.46	−1.42
2	b	1.36	1.45	1.52	1.48	1.48
3	r	0.9125	0.9359	0.9315	0.9904	0.9962
4	p(r)	5.99 × 10^−4^	6.27 × 10^−4^	2.27 × 10^−3^	2.87 × 10^−7^	1.33 × 10^−7^
5	LD	1.0891	0.9194	0.5928	0.2987	0.1666
6	SSE	1.34	9.90 × 10^−1^	9.22 × 10^−1^	1.54 × 10^−1^	5.95 × 10^−2^
7	SEE	4.37 × 10^−1^	4.06 × 10^−1^	4.30 × 10^−1^	1.48 × 10^−1^	9.96 × 10^−2^
2. Cross-validation method (CVM)						
8	SSEcv	2.40	1.95	2.22	2.34 × 10^−1^	9.72 × 10^−2^
9	SEEcv	5.86 × 10^−1^	5.70 × 10^−1^	6.67 × 10^−1^	1.83 × 10^−1^	1.27 × 10^−1^
3. ANOVA analysis of the regression model						
10	F	34.8	42.3	32.8	358	792
11	p(ANOVA)	5.99 × 10^−4^	6.27 × 10^−4^	2.27 × 10^−3^	2.87 × 10^−7^	1.33 × 10^−7^
4. Testing of the regression model coefficients						
12	p(a)	1.62 × 10^−3^	1.54 × 10^−3^	5.64 × 10^−3^	9.36 × 10^−7^	4.79 × 10^−7^
13	p(b)	5.99 × 10^−4^	6.27 × 10^−4^	2.27 × 10^−3^	2.87 × 10^−7^	1.33 × 10^−7^

Simple linear regression model: a, b: Intercept and slope of the regression line, r, p(r): Correlation coefficient, LD: Detection limit of melamine, SSE, SEE: Sum of squares of residuals and standard error of estimate. Cross-validation: F, p(ANOVA): F-statistic and associated *p*-value for model significance. Regression coefficients testing: p(a), p(b): t-statistics and *p*-values for intercept and slope.

## Data Availability

Data is unavailable due to privacy.

## Supplementary Materials

The following supporting information can be downloaded at: https://www.mdpi.com/article/10.3390/molecules31111912/s1, Table S1: Proximate analysis of brown rice flour investigated; Figure S1. Workflow illustrating the main sources of spectral variability in ATR–FTIR analysis of brown rice flour and the role of spectral normalization in improving the reliability of melamine detection. Table S2. Characterization of linear regression models obtained before and after the exclusion of outlier data. Table S3. The characterization of these regression models associated to PCA analysis.

## Abbreviations

The following abbreviations are used in this manuscript:

FTIR	Fourier Transform Infrared
PCA	Principal Component Analysis
PC1	The first principal component
PC2	The second principal component
R^2^	Correlation coefficient
Me	Melamine
BRF	Brown rice flour
U-BRF	Unadulterated brown rice flour
RCD	Relative crystallinity degree
RSD	Relative standard deviation
