# Supplementary material for: FTIR Spectroscopy Coupled with Principal Component Analysis for Rapid Screening of Melamine Adulteration in Brown Rice Flour
Source: Molecules. 2026 Jun 2;31(11):1912. doi: 10.3390/molecules31111912 (PMC13258603; doi:10.3390/molecules31111912)
Supplement: Supplementary file 1 [file molecules-31-01912-s001.zip › molecules-4315254-supplementary.pdf]

# FTIR Spectroscopy Coupled with Principal Component Analysis for Rapid Screening of Melamine Adulteration in Brown Rice Flour

**Cristina Pintilii, Leonard Mihaly Cozmuta, Zsolt Szakacs and Anca Mihaly Cozmuta ***

Chemistry-Biology Department, Faculty of Science, North University Center of Baia Mare, Technical University of Cluj-Napoca, Victoriei Str. 76, 430122 Baia Mare, Romania; pintiliicristi@yahoo.com (C.P.); mihalyleonard@yahoo.com (L.M.C.); szakacsz@yahoo.com (Z.S.)

* Correspondence: anacamihalycozmuta@gmail.com; Tel.: +40-741949669

## S1. SM. Materials and methods

### *S1.1. SM. Proximate analysis of brown rice flour* (Pintilii et al., 2025)

**Table S1.** Proximate analysis of brown rice flour investigated

| Parameter | Brown rice flour |
|---|---|
| Moisture, % | 10.78 ± 0.13 |
| Ash, % | 1.41 ± 0.10 |
| Protein, % | 8.33 ± 0.12 |
| Fat, % | 2.23 ± 0.12 |
| Carbohydrates, % | 76.80 ± 0.24 |
| Fiber, % | 0.44 ± 0.105 |
| Total polyphenols, mg GAE/Kg | 1288.11 ± 40.70 |

Data are presented as mean ± standard deviation (n = 3).

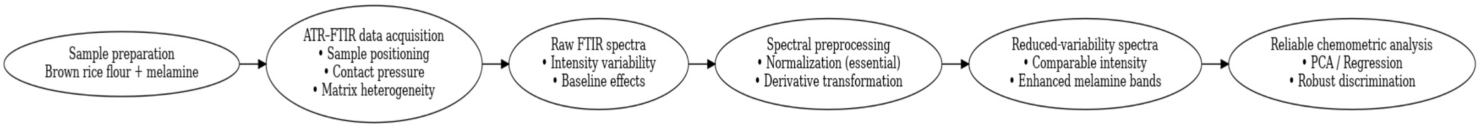


***Figure S1.*** *Workflow illustrating the main sources of spectral variability in ATR–FTIR analysis of brown rice flour and the role of spectral normalization in improving the reliability of melamine detection.*

**S2. Application of FTIR for the identification of melamine adulteration in brown rice flour**

**Table S2.** Characterization of linear regression models obtained before and after the exclusion of outlier data.

| | **Model** | **M1** | **M2** | **M3** | **M4** | **M5** | **M6** | **M7** | **M8** | **M9** |
|---|---|---|---|---|---|---|---|---|---|---|
| | **Wavenumber, $cm^{-1}$** | **3466** | **3466** | **3415** | **3415** | **1431** | **1431** | **1431** | **810** | **810** |
| **1. Simple linear regression model** | | | | | | | | | | |
| 1 | n | 9 | 7 | 9 | 7 | 9 | 8 | 7 | 9 | 8 |
| 2 | a | -1.14 E-06 | -1.24 E-06 | 6.77 E-06 | 5.59 E-06 | -4.81 E-05 | -5.15 E-05 | -5.42 E-05 | -1.25 E-04 | -1.21 E-04 |
| 3 | b | 4.96 E-05 | 4.93 E-05 | 2.99 E-05 | 3.13 E-05 | 4.24 E-05 | 4.43 E-05 | 4.60 E-05 | 9.83 E-05 | 9.83 E-05 |
| 4 | **r** | **0.9935** | **0.9964** | **0.9903** | **0.9973** | **0.9717** | **0.9831** | **0.9831** | **0.9794** | **0.9909** |
| 5 | t(r) | 23.0727 | 26.2633 | 18.8231 | 30.5602 | 10.8860 | 13.1670 | 12.0124 | 12.8453 | 18.0773 |
| 6 | p(r) | 7.28 E-08 | 1.50 E-06 | 2.97 E-07 | 7.04 E-07 | 1.22 E-05 | 1.18 E-05 | 7.05 E-05 | 4.02 E-06 | 1.84 E-06 |
| 7 | R^2 | 0.9870 | 0.9928 | 0.9806 | 0.9947 | 0.9442 | 0.9665 | 0.9665 | 0.9593 | 0.9820 |
| 8 | Adj(R^2) | 0.9852 | 0.9914 | 0.9779 | 0.9936 | 0.9363 | 0.9610 | 0.9598 | 0.9535 | 0.9790 |
| 9 | **LD** | **0.4297** | **0.1408** | **0.5814** | **0.1484** | **0.7040** | **0.6036** | **0.2948** | **0.4406** | **0.2641** |
| 10 | **x(rec)%** | **97.0130** | **98.9910** | **104.9570** | **100.2512** | **106.0627** | **104.7916** | **99.3289** | **98.9120** | **97.8510** |
| 11 | **ERMx(%)** | **8.9848** | **3.5063** | **10.7248** | **4.0512** | **16.3645** | **13.4560** | **7.2141** | **11.4446** | **6.2886** |
| 12 | **ERMy(%)** | **11.6651** | **3.7168** | **6.5633** | **3.4371** | **-11.8943** | **2.4994** | **10.0411** | **-13.0299** | **-13.9246** |
| 13 | **SSE** | **1.16 E-10** | **5.04 E-11** | **6.38 E-11** | **1.55 E-11** | **3.83 E-10** | **2.26 E-10** | **1.91 E-10** | **1.48 E-09** | **6.39 E-10** |
| 14 | **SEE** | **4.08 E-06** | **3.18 E-06** | **3.02 E-06** | **1.76 E-06** | **7.40 E-06** | **6.14 E-06** | **6.18 E-06** | **1.45 E-05** | **1.03 E-05** |
| **2. Cross-validation method (CVM)** | | | | | | | | | | |
| 15 | x(rec)%cv | 95.5437 | 98.7003 | 107.0478 | 100.5069 | 107.5382 | 107.0924 | 98.9771 | 99.9547 | 98.1173 |
| 16 | ERMx(%)cv | 11.5392 | 4.6300 | 13.5837 | 5.2041 | 19.8635 | 17.8060 | 9.2555 | 14.1587 | 7.9353 |
| 17 | ERMy(%)cv | 15.1146 | 4.8818 | 8.2814 | 4.3993 | 33.9121 | 29.7291 | 32.3518 | 30.5834 | 25.6189 |
| 18 | SSEcv | 1.78 E-10 | 9.16 E-11 | 1.00 E-10 | 2.65 E-11 | 6.17 E-10 | 3.88 E-10 | 3.31 E-10 | 2.41 E-09 | 1.14 E-09 |

| | | | | | | | | | | |
|---|---|---|---|---|---|---|---|---|---|---|
| 19 | SEEcv | 5.04 E-06 | 4.28 E-06 | 3.78 E-06 | 2.30 E-06 | 9.39 E-06 | 8.04 E-06 | 8.14 E-06 | 1.85 E-05 | 1.38 E-05 |
| **3. ANOVA analysis of the regression model** | | | | | | | | | | |
| 20 | SSR | 8.84 E-09 | 6.96E-09 | 3.23E-09 | 2.90E-09 | 6.48E-09 | 6.54E-09 | 5.52E-09 | 3.48E-08 | 3.48E-08 |
| 21 | SSE | 1.16 E-10 | 5.04E-11 | 6.38E-11 | 1.55E-11 | 3.83E-10 | 2.26E-10 | 1.91E-10 | 1.48E-09 | 6.39E-10 |
| 22 | SST | 8.96 E-09 | 7.01 E-09 | 3.29 E-09 | 2.92 E-09 | 6.87 E-09 | 6.77 E-09 | 5.71 E-09 | 3.63 E-08 | 3.55 E-08 |
| 23 | df(r) | 1 | 1 | 1 | 1 | 1 | 1 | 1 | 1 | 1 |
| 24 | df(e) | 7 | 5 | 7 | 5 | 7 | 6 | 5 | 7 | 6 |
| 25 | df(t) | 8 | 6 | 8 | 6 | 8 | 7 | 6 | 8 | 7 |
| 26 | MS(r) | 8.84 E-09 | 6.96E -09 | 3.23 E-09 | 2.90 E-09 | 6.48 E-09 | 6.54 E-09 | 5.52 E-09 | 3.48 E-08 | 3.48 E-08 |
| 27 | MS(e) | 1.66 E-11 | 1.01 E-11 | 9.12 E-12 | 3.11 E-12 | 5.47 E-11 | 3.77 E-11 | 3.82 E-11 | 2.11 E-10 | 1.07 E-10 |
| 28 | F | 5.32 E+02 | 6.90 E+02 | 3.54 E+02 | 9.34 E+02 | 1.19 E+02 | 1.73 E+02 | 1.44 E+02 | 1.65 E+02 | 3.27 E+02 |
| 29 | **p(ANOVA)** | **7.28 E-08** | **1.50 E-06** | **2.97 E-07** | **7.04 E-07** | **1.22 E-05** | **1.18 E-05** | **7.05 E-05** | **4.02 E-06** | **1.84 E-06** |
| **4. Testing of the regression model coefficients** | | | | | | | | | | |
| 30 | Sy/x | 4.08 E-06 | 3.18 E-06 | 3.02 E-06 | 1.76 E-06 | 7.40 E-06 | 6.14 E-06 | 6.18 E-06 | 1.45 E-05 | 1.03 E-05 |
| 31 | Sa | 2.52 E-06 | 2.43 E-06 | 1.86 E-06 | 1.27 E-06 | 4.57 E-06 | 4.14 E-06 | 5.03 E-06 | 8.97 E-06 | 6.49 E-06 |
| 32 | Sb | 2.15 E-06 | 1.88 E-06 | 1.59 E-06 | 1.02 E-06 | 3.90 E-06 | 3.36 E-06 | 3.83 E-06 | 7.65 E-06 | 5.44 E-06 |
| 33 | t(a) | 4.52 E-01 | 5.12 E-01 | 3.63 E+00 | 4.40 E+00 | 1.05 E+01 | 1.24 E+01 | 1.08 E+01 | 1.39 E+01 | 1.87 E+01 |
| 34 | t(b) | 2.31 E+01 | 2.63 E+01 | 1.88 E+01 | 3.06 E+01 | 1.09 E+01 | 1.32 E+01 | 1.20 E+01 | 1.28 E+01 | 1.81 E+01 |
| 35 | **p(a)** | **6.65** | **6.30** | **8.40** | **7.02** | **1.52** | **1.66** | **1.19** | **2.35** | **1.51** |

| | | | | | | | | | | |
|---|---|---|---|---|---|---|---|---|---|---|
| | | E-01 | E-01 | E-03 | E-03 | E-05 | E-05 | E-04 | E-06 | E-06 |
| 36 | **p(b)** | **7.28 E-08** | **1.50 E-06** | **2.97 E-07** | **7.04 E-07** | **1.22 E-05** | **1.18 E-05** | **7.05 E-05** | **4.02 E-06** | **1.84 E-06** |

Simple linear regression model: n: Number of observations, a, b: Intercept and slope of the regression line, r, t(r), p(r): Correlation coefficient, its t-statistic, and associated p-value, $R^2$, Adj($R^2$): Coefficient of determination and adjusted $R^2$, LD: Detection limit of melamine, x(rec)%, ERMx(%), ERMy(%): Recovered concentration (%), relative errors in x and y, SSE, SEE: Sum of squares of residuals and standard error of estimate. Cross-validation: x(rec)%cv, ERMx(%)cv, ERMy(%)cv, SSEcv, SEEcv: Same as above, calculated during cross-validation to evaluate predictive accuracy. ANOVA for regression: SSR, SSE, SST: Sum of squares for regression, error, and total (SST=SSR+SSE), df(r), df(e), df(t): Degrees of freedom for regression, error, and total, MS(r), MS(e): Mean squares for regression and error, F, p(ANOVA): F-statistic and associated p-value testing model significance. Testing regression coefficients: Sy/x, Sa, Sb: Standard errors of estimate, intercept, and slope, t(a), t(b), p(a), p(b): t-statistics and p-values for intercept and slope.

**Table S3.** The characterization of these regression models associated to PCA analysis.

| | **Model** | **M1** | **M2** | **M3** | **M4** | **M5** |
|---|---|---|---|---|---|---|
| | | **Absorbances** | **Absorbances** | **Absorbances** | **Second-order derivatives** | **Second-order derivatives** |
| | | **PC2=f(C)** | **PC2=f(C)** | **PC2=f(C)** | **PC1=f(C)** | **PC1=f(C)** |
| **1. Simple linear regression model** | | | | | | |
| 1 | n | 9 | 8 | 7 | 9 | 8 |
| 2 | a | -1.34 E+00 | -1.50 E+00 | -1.62 E+00 | -1.46 E+00 | -1.42 E+00 |
| 3 | b | 1.36 E+00 | 1.45 E+00 | 1.52 E+00 | 1.48 E+00 | 1.48 E+00 |
| 4 | r | 0.9125 | 0.9359 | 0.9315 | 0.9904 | 0.9962 |
| 5 | t(r) | 5.9005 | 6.5074 | 5.7254 | 18.9116 | 28.1377 |
| 6 | p(r) | 5.99 E-04 | 6.27 E-04 | 2.27 E-03 | 2.87 E-07 | 1.33 E-07 |
| 7 | R^2 | 0.8326 | 0.8759 | 0.8677 | 0.9808 | 0.9925 |
| 8 | Adj(R^2) | 0.8087 | 0.8552 | 0.8412 | 0.9781 | 0.9912 |
| 9 | **LD** | **1.0891** | **0.9194** | **0.5928** | **0.2987** | **0.1666** |
| 10 | **x(rec)%** | **106.9783** | **104.8267** | **97.3216** | **99.5128** | **98.8036** |
| 11 | **ERMx(%)** | **26.8568** | **23.4448** | **16.5927** | **7.7663** | **4.0814** |
| 12 | **ERMy(%)** | **-77.6704** | **-32.5229** | **-34.5850** | **-5.0105** | **9.7169** |
| 13 | **SSE** | **1.34** | **9.90** | **9.22** | **1.54** | **5.95** |

| | | | | | | |
|---|---|---|---|---|---|---|
| | | **E+00** | **E-01** | **E-01** | **E-01** | **E-02** |
| 14 | **SEE** | **4.37 E-01** | **4.06 E-01** | **4.30 E-01** | **1.48 E-01** | **9.96 E-02** |
| **2. Cross-validation method (CVM)** | | | | | | |
| 15 | x(rec)%cv | 111.4445 | 110.4540 | 100.1228 | 100.1572 | 98.8931 |
| 16 | ERMx(%)cv | 33.9367 | 31.8675 | 22.8159 | 9.4996 | 5.0237 |
| 17 | ERMy(%)cv | 153.2607 | 104.0176 | 117.6007 | 31.5211 | 20.5576 |
| 18 | SSEcv | 2.40 E+00 | 1.95 E+00 | 2.22 E+00 | 2.34 E-01 | 9.72 E-02 |
| 19 | SEEcv | 5.86 E-01 | 5.70 E-01 | 6.67 E-01 | 1.83 E-01 | 1.27 E-01 |
| **3. ANOVA analysis of the regression model** | | | | | | |
| 20 | SSR | 6.66 E+00 | 6.99 E+00 | 6.05 E+00 | 7.85 E+00 | 7.85 E+00 |
| 21 | SSE | 1.34 E+00 | 9.90 E-01 | 9.22 E-01 | 1.54 E-01 | 5.95 E-02 |
| 22 | SST | 8.00 E+00 | 7.98 E+00 | 6.97 E+00 | 8.00 E+00 | 7.91 E+00 |
| 23 | df(r) | 1 | 1 | 1 | 1 | 1 |
| 24 | df(e) | 7 | 6 | 5 | 7 | 6 |
| 25 | df(t) | 8 | 7 | 6 | 8 | 7 |
| 26 | MS(r) | 6.66 E+00 | 6.99 E+00 | 6.05 E+00 | 7.85 E+00 | 7.85 E+00 |
| 27 | MS(e) | 1.91 E-01 | 1.65 E-01 | 1.84 E-01 | 2.19 E-02 | 9.91 E-03 |
| 28 | F | 3.48 E+01 | 4.23 E+01 | 3.28 E+01 | 3.58 E+02 | 7.92 E+02 |
| 29 | **p(ANOVA)** | **5.99 E-04** | **6.27 E-04** | **2.27 E-03** | **2.87 E-07** | **1.33 E-07** |
| **4. Testing of the regression model coefficients** | | | | | | |

| | | | | | | |
|---|---|---|---|---|---|---|
| 30 | Sy/x | 4.37 E-01 | 4.06 E-01 | 4.30 E-01 | 1.48 E-01 | 9.96 E-02 |
| 31 | Sa | 2.70 E-01 | 2.74 E-01 | 3.49 E-01 | 9.15 E-02 | 6.26 E-02 |
| 32 | Sb | 2.30 E-01 | 2.23 E-01 | 2.66 E-01 | 7.80 E-02 | 5.25 E-02 |
| 33 | t(a) | 4.97 E+00 | 5.48 E+00 | 4.64 E+00 | 1.59 E+01 | 2.27 E+01 |
| 34 | t(b) | 5.90 E+00 | 6.51 E+00 | 5.73 E+00 | 1.89 E+01 | 2.81 E+01 |
| 35 | **p(a)** | **1.62 E-03** | **1.54 E-03** | **5.64 E-03** | **9.36 E-07** | **4.79 E-07** |
| 36 | **p(b)** | **5.99 E-04** | **6.27 E-04** | **2.27 E-03** | **2.87 E-07** | **1.33 E-07** |

Simple linear regression model: n: Number of observations, a, b: Intercept and slope of the regression line, r, t(r), p(r): Correlation coefficient, its t-statistic, and associated p-value, $R^2$, Adj($R^2$): Coefficient of determination and adjusted $R^2$, LD: Detection limit of melamine, x(rec)%, ERMx(%), ERMy(%): Recovered concentration (%), relative errors in x and y, SSE, SEE: Sum of squares of residuals and standard error of estimate. Cross-validation: x(rec)%cv, ERMx(%)cv, ERMy(%)cv, SSEcv, SEEcv: Same as above, calculated during cross-validation to assess predictive accuracy, ANOVA: SSR, SSE, SST: Regression, error, and total sum of squares (SST = SSR + SSE), df(r), df(e), df(t): Degrees of freedom for regression, error, and total, MS(r), MS(e): Mean squares for regression and error, F, p(ANOVA): F-statistic and associated p-value for model significance. Regression coefficients testing: Sy/x, Sa, Sb: Standard errors of estimate, intercept, and slope, t(a), t(b), p(a), p(b): t-statistics and p-values for intercept and slope.
